# Uranium–nitride chemistry: uranium–uranium electronic communication mediated by nitride bridges†‡

**DOI:** 10.1039/d2dt00998f

**Published:** 2022-05-20

**Authors:** David M. King, Benjamin E. Atkinson, Lucile Chatelain, Matthew Gregson, John A. Seed, Ashley J. Wooles, Nikolas Kaltsoyannis, Stephen T. Liddle

**Affiliations:** School of Chemistry, University of Nottingham, University Park Nottingham NG7 2RD UK; Department of Chemistry, University of Manchester Oxford Road Manchester M13 9PL UK steve.liddle@manchester.ac.uk nikolas.kaltsoyannis@manchester.ac.uk

## Abstract

Treatment of [U^IV^(N_3_)(Tren^TIPS^)] (1, Tren^TIPS^ = {N(CH_2_CH_2_NSiPr^i^_3_)_3_}^3−^) with excess Li resulted in the isolation of [{U^IV^(μ-NLi_2_)(Tren^TIPS^)}_2_] (2), which exhibits a diuranium(iv) ‘diamond-core’ dinitride motif. Over-reduction of 1 produces [U^III^(Tren^TIPS^)] (3), and together with known [{U^V^(μ-NLi)(Tren^TIPS^)}_2_] (4) an overall reduction sequence 1 → 4 → 2 → 3 is proposed. Attempts to produce an odd-electron nitride from 2 resulted in the formation of [{U^IV^(Tren^TIPS^)}_2_(μ-NH)(μ-NLi_2_)Li] (5). Use of heavier alkali metals did not result in the formation of analogues of 2, emphasising the role of the high charge-to-radius-ratio of lithium stabilising the charge build up at the nitride. Variable-temperature magnetic data for 2 and 5 reveal large low-temperature magnetic moments, suggesting doubly degenerate ground states, where the effective symmetry of the strong crystal field of the nitride dominates over the spin–orbit coupled nature of the ground multiplet of uranium(iv). Spin Hamiltonian modelling of the magnetic data for 2 and 5 suggest U⋯U anti-ferromagnetic coupling of −4.1 and −3.4 cm^−1^, respectively. The nature of the U⋯U electronic communication was probed computationally, revealing a borderline case where the prospect of direct uranium–uranium bonding was raised, but in-depth computational analysis reveals that if any uranium–uranium bonding is present it is weak, and instead the nitride centres dominate the mediation of U⋯U electronic communication. This highlights the importance of obtaining high-level *ab initio* insight when probing potential actinide–actinide electronic communication and bonding in weakly coupled systems. The computational analysis highlights analogies between the ‘diamond-core’ dinitride of 2 and matrix-isolated binary U_2_N_2_.

## Abstract 2

Sequential reduction of a uranium-azide through all the nitride steps is revealed. Experimentally determined U–U coupling in the diuranium(iv)–dinitride is shown by *ab initio* calculations to stem from the nitride bridges, not U–U bonding.

## Graphical abstract

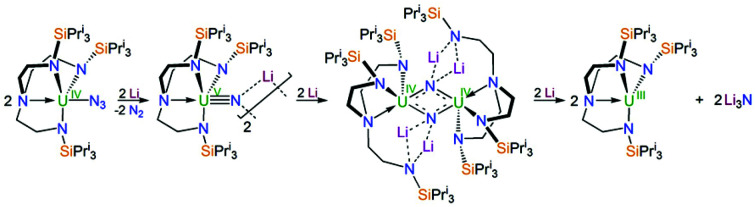

## Introduction

In recent times there has been increasing interest in molecular early actinide nitride complexes^1^ due to fundamental questions about their bonding,^2^ reactivity,^3^ magnetism,^2*a,c*,3*f,i*,*j*^ and potential relevance to materials science.^4^ The quest for isolable, molecular terminal uranium–nitrides was accomplished in 2012 with the synthesis of [U^V^(N)(Tren^TIPS^)][Na(12C4)_2_] (Tren^TIPS^ = {N(CH_2_CH_2_NSiPr^i^_3_)_3_}^3−^; 12C4 = 12-crown-4 ether) and then [U^VI^(N)(Tren^TIPS^)] in 2013,^5^ and since then relatively few uranium(v) and (vi) terminal nitrides have been isolated.^2*a*,6^ In contrast, isolable bridging nitride complexes of the early actinides are far more numerous.^1^ For mid (+4) and low (+3) oxidation state uranium nitrides the bonding motif is overwhelmingly that of a bridging mono-nitride, *e.g.* {X_*n*_UNUX_*n*_} or {X_*n*_UNA} (X = monoanionic donor centre, mono- or polydentate ligands; A = Lewis acid acceptor group),^7^ whereas for high (+5, +6) oxidation states of uranium the bridging ‘diamond-core’ dinitride motif {X_*n*_U(μ-N)_2_UX_*n*_} is usually found.^8^ To date, however, mid- or low-oxidation state ‘diamond-core’ dinitrides are rare, being restricted to trivalent binary U^III^_2_N_2_ studied in cryogenic matrix isolation experiments,^9^ a molecular tetrauranium(iv)–dirhodium cluster,^10^ a mixed-valent diuranium(IV/V)–calix[4]tetrapyrrole complex,^11^ and a tetrauranium(iv) cluster.^12^ There are, however, no examples of diuranium(iv) complexes exhibiting the ‘diamond-core’ dinitride motif.

Since Tren^TIPS^ has proven effective at stabilising terminal nitride linkages at uranium(v) and (vi), and other novel linkages with actinides in the +4 oxidation state,^13^ we wondered whether the (iv) analogue could also be accessed. Here, we report that efforts employing a reductive approach have resulted in the isolation of a dinuclear uranium(iv)–nitride–dilithium complex with a ‘diamond-core’ dinitride structural motif. This complex permits us to recognise a logical reduction sequence of azide to nitride, thus rationalising prior experimental observations. Efforts to abstract the lithium ions result in conversion of the dinitride to a nitride-imide, emphasising the stabilising role of the lithium cations and also the rather polarised nature of uranium(iv)–nitride bonds. Variable-temperature magnetism studies reveal modest anti-ferromagnetic (AF) coupling, experimentally establishing uranium–uranium electronic communication. Quantum chemical computational efforts to rationalise this electronic communication initially raised the possibility of uranium–uranium bonding, which would be a significant finding,^14^ but in-depth computational analysis reveals that if any uranium–uranium bonding is present it is rather weak, and instead the nitride centres dominate the mediation of the electronic communication between the uranium(iv) ions. Our computational results emphasise the importance of obtaining high-level *ab initio* insight when probing potential actinide–actinide electronic communication and bonding in weakly coupled systems. This computational analysis highlights analogies between the ‘diamond-core’ dinitride reported here and matrix-isolated binary U_2_N_2_, potentially providing conceptual links between microscopic inert matrix and isolable macroscopic species.

## Results and discussion

### Synthesis of the dinuclear nitride and nitride–imide complexes 2 and 5

**Scheme 1:**
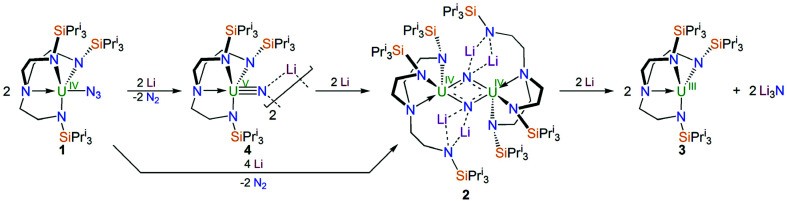
Synthesis of 2 from 1 and/or 4 and eventual over-reduction to 3 and Li_3_N.

Treatment of a toluene solution of [U^IV^(N_3_)(Tren^TIPS^)] (1)^5*b*^ with excess lithium over 5 days with stirring and occasional sonication resulted in the precipitation of a dark red solid; isolation of this red solid by filtration and extraction into boiling toluene and cooling resulted in the isolation of the red crystalline nitride complex [{U^IV^(μ-NLi_2_)(Tren^TIPS^)}_2_] (2) in yields typically averaging 58%, Scheme 1. The initial precipitation of the dark red solid is also accompanied by the solution turning dark blue, which is characteristic of the formation of [U^III^(Tren^TIPS^)] (3)^5*a*^ by over-reduction of 1 (and by implication 2). The formation of 3 was confirmed by ^1^H NMR spectroscopy and a unit cell check on dark blue crystals obtained from one reaction, and whilst the formation of Li_3_N was not confirmed due to the presence of excess Li powder and precipitated 2 its formation seems assured on mass-balance grounds. Seeking enhanced control of this reaction, we examined the reduction of a toluene solution of the previously reported nitride complex [{U^V^(μ-NLi)(Tren^TIPS^)}_2_] (4)^2*a*^ with Li powder and over a 5 day stir with occasional sonication, and observed the same outcome, namely a dark red precipitate and dark blue solution, which after work-up produced 2 in essentially the same yield, Scheme 1. Thus, we deduce that 1 is reduced stepwise to 4 then 2, and ultimately is reduced all the way to 3 and Li_3_N. This is consistent with the fact that whilst the terminal nitride linkage can be formed and retained at uranium(v/vi) using Tren^TIPS^ it is a linkage that has also been found to be readily displaced.^5*b*^ Certainly, on hard–soft-acid–base grounds N^3−^ would be predicted to be a better match with high rather than intermediate oxidation states of uranium which is reflected by the observations here and also in the highly reactive nature of diuranium(iii)–nitride complexes more generally.^3*f*,7*i*^

**Scheme 2:**
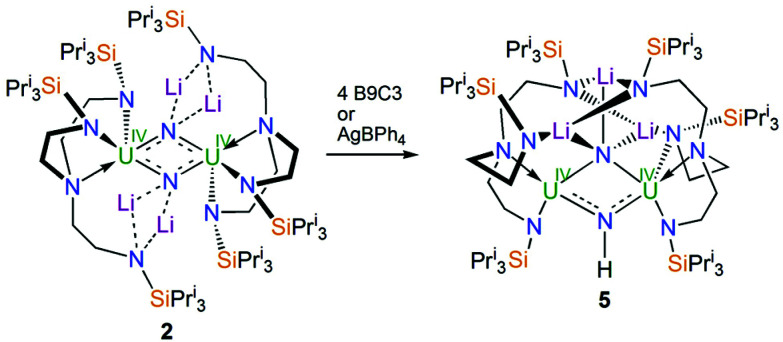
Synthesis of 5 from 2. B9C3 = benzo-9-crown-3 ether. The unidentified by-products are not shown.

With 2 available reliably, we sought to examine the abstraction of the Li ions, and also its oxidation. Indeed, since the reaction sequence 1 → 4 → 2 → 3 introduces even numbers of Li ions and electrons we were interested to ascertain whether odd-numbered combinations would be accessible. However, treatment of 2 with benzo-9-crown-3 ether or AgBPh_4_ consistently both gave the same outcome, which is formation of a red solid; recrystallisation of this red product afforded red crystals of the nitride–imide complex [{U^IV^(Tren^TIPS^)}_2_(μ-NH)(μ-NLi_2_)Li] (5) in yields ranging from 13 to 53%, Scheme 2. Though the formation of 5 is a single example, by abstraction and/or oxidation the result is that one of the nitrides acquires a proton and converts to an imide, suggesting that odd-numbered Li ion/electron count species are less stable than the corresponding even numbered complexes for uranium–Tren^TIPS^. Also, this likely reflects the not so favourable combination of uranium(iv) with an otherwise terminal nitride (compared to uranium(v/vi)). Hence, polar uranium(iv)–nitride linkages would be destabilised by removal of stabilising Li ions. Germane to this point, analogous reactivity has been observed with a tetrameric uranium(iv)–arsenido,^13*e*^ which clearly exhibits highly polarised U–As bonds, where attempts to remove the stabilising K ions resulted in the arsenido acquiring a proton and converting to an arsinidene. We note in passing that treatment of 1 or 4M (M = Li–Cs) with excess quantities of heavier alkali metals (Na–Cs or MC_8_, M = K–Cs) results in either the isolation of the heavier alkali metal analogues of 4, or with extended reaction times extensive decomposition. Attempts to reduce 1 with two equivalents of alkali metal resulted in slower reactions that only produced 4M and then ultimately decomposed under extended stirring, implying limiting kinetic factors. This implies that lithium in particular, with its high charge-to-radius-ratio, is essential to isolating 2 due to the stabilisation of the nitride charge by the strongly polarising lithium centres. This effect would be expected to diminish with heavier alkali metals, resulting in more destabilised uranium–nitride linkages.

### Solid-state structures of the dinuclear nitride and nitride–imide complexes 2 and 5

**Fig. 1:**
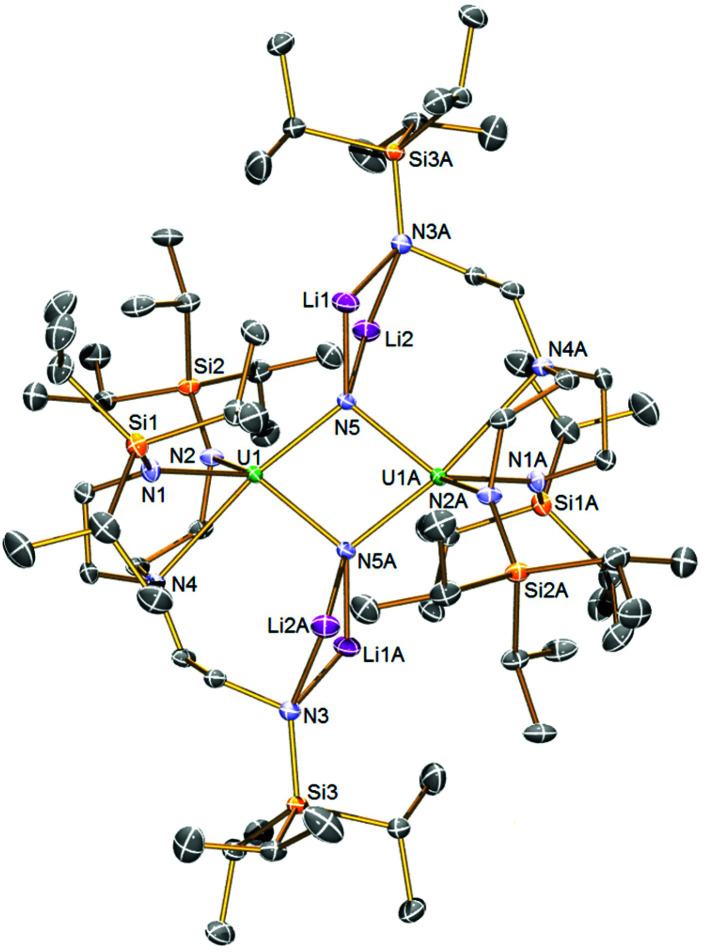
Solid-state molecular structures of 2 at 120 K with displacement ellipsoids set to 40%. Hydrogen atoms are omitted for clarity.

The solid-state structure of 2 was determined by single-crystal X-ray diffraction, Fig. 1. Complex 2 is a dinuclear assembly of two {U^IV^(μ-N)(Tren^TIPS^)}^2−^ units where the central U_2_N_2_ ring resides over a crystallographic inversion centre (U⋯U = 3.4044(2) Å). Each nitride is four-coordinate, being each bound to two lithium ions in addition to the two uranium centres. The lithium ions are then bonded to an amide from one of the Tren^TIPS^ arms in butterfly Li_2_N_2_ four-membered rings, which is an exceptional occurrence since all other uranium–Tren^TIPS^ complexes exhibit the conventional *C*_3_ coordination mode where all three amides are bound to the same uranium ion. The departure of one Tren^TIPS^ amide arm from the coordination sphere of each uranium opens up a vacant coordination site which has then been filled by a bridging nitride. Formally, the lithium cations are two-coordinate, but, in reality, their coordination spheres are supplemented by numerous Li⋯HC interactions with the TIPS Pr^i^ groups. The U1–N5 and U1–N4 distances are 2.153(2) and 2.776(2) Å, respectively. For the nitride this reflects the approximately *trans* nature of this linkage (N5–U1–N4 ∠ = 169.22(7)°), and also that the uranium oxidation state is +4 so the U–N bonds will be longer than uranium +5 and +6 derivatives. Also, it is likely that a *trans*-influence operates in 2 whereas in [U^VI^(N)(Tren^TIPS^)] both the U–N_nitride_ (∼1.8 Å) and U–N_amine_ (∼2.5 Å) distances are short suggesting the presence of an inverse-*trans*-influence.^5*b*^ The U1–N5A distance is 2.208(2) Å, rendering the core U_2_N_2_ ring slightly asymmetric. Unsurprisingly, the U–N_nitride_ distances in 2 are ∼0.15–0.2 Å longer than diuranium–nitride complexes with only one bridging nitride, but are generally consistent with other poly-nitride actinide complexes.^7^ We also note that that U–N distances in 2 are slightly longer overall than in U_2_N_2_ ‘diamond-core’ complexes of uranium(v) and(vi).^8*b*^ The U–N_amide_ distances of 2.369(2) and 2.348(2) Å are slightly longer than is typical for uranium(iv)–Tren^TIPS^ complexes (∼2.25 Å), which likely reflects the sterically congested nature of 2 and the presence of two nitrides, albeit bridging in nature, in the coordination sphere of each uranium ion. The Li–N distances are unremarkable.

**Fig. 2:**
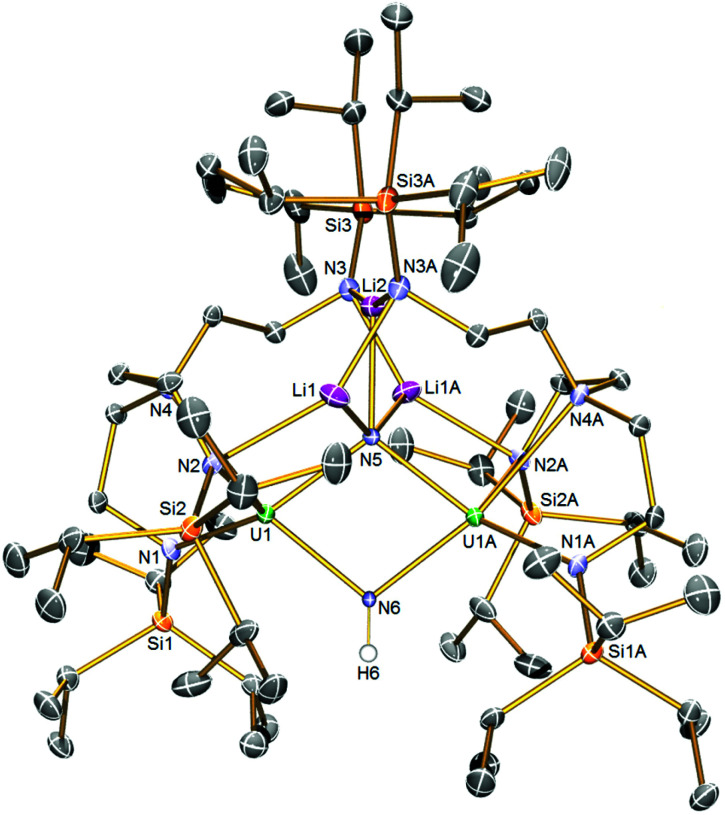
Solid-state molecular structure of 5 at 120 K with displacement ellipsoids set to 40%. Non-imide Hydrogen atoms are omitted for clarity.

The solid-state structure of 5 is illustrated in Fig. 2. Like 2, complex 5 is built around a central U_2_N_2_ ring, however this time the ring resides on a crystallographic two-fold rotation axis that bisects the N⋯N vector, which renders the two uranium-containing halves of the molecule equivalent by symmetry simplifying the resulting discussion. The U⋯U distance is 3.4611(4) Å, betraying the presence of an imide as well as nitride ligand. The N6 atom is assigned as an imide, on grounds of bond lengths, charge balance, and calculations (see below). The N5 atom is a nitride, and this centre binds to three lithium atoms which, in contrast to the even distribution of lithium atoms in 2, all reside on one side of 5 disposed on or around the two-fold rotation axis. The lithium atoms in 5 are then further coordinated by Tren^TIPS^ amide arms, in two instances to amides still bound to uranium and, for the central lithium to amides that have no contact to uranium, as in 2. The U1–N5 distance is 2.171(4) Å, which is only marginally distinguishable from the analogous distance in 2 by the 3σ-criterion, but the U1–N6 distance is longer at 2.212(4) Å reflecting its protonated and thence imide not nitride status. Again, the U–N_amide_ distances of 2.329(5) and 2.377(5) Å and the U1–N4 distance of 2.697(5) Å are consistent with uranium(iv)–N distances in such sterically crowded molecules. The Li–N distances are unremarkable.

### Characterisation of the dinuclear nitride and nitride-imide complexes 2 and 5

**Fig. 3:**
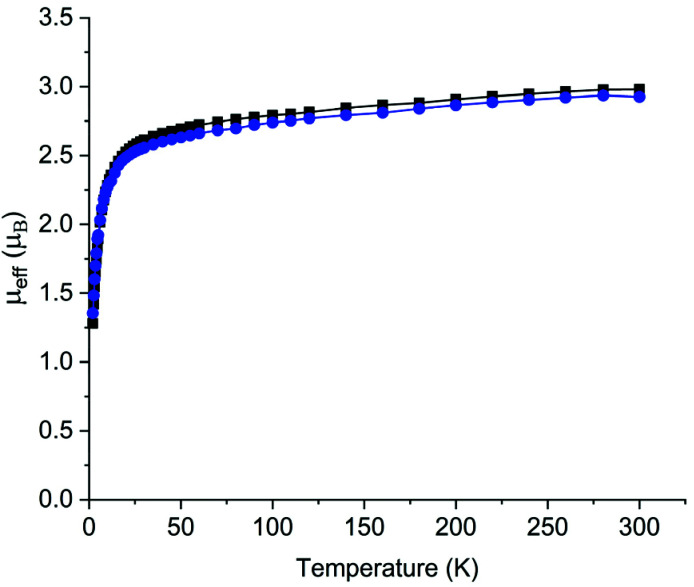
Variable-temperature effective magnetic moment (*μ*_eff_) *versus* temperature for 2 (black squares) and 5 (blue circles), per U ion. The lines are a guide to the eye only, since the spin Hamiltonian fits data for the whole molecule (see ESI‡) rather than per ion.

Once recrystallised, 2 and 5 are insoluble in aromatic solvents and decompose rapidly in polar solvents and thus NMR and UV/Vis/NIR spectra were impracticable to obtain. ATR-IR and elemental analyses are consistent with the proposed formulations of 2 and 5, and for the latter a broad absorption centred at 3395 cm^−1^ is assigned as the bridging imide N–H stretch due to its similarity to the N–H stretches of 3390, 3393, and 3397 cm^−1^ for [{Th(Tren^DMBS^)}_2_(μ-NH)] (Tren^DMBS^ = {N(CH_2_CH_2_NSiMe_2_Bu^t^)_3_}^3−^),^2*c*^ [{Th(Tren^TIPS^)(μ-NHLi)}_2_], and [{Th(Tren^TIPS^)(μ-NHNa)}_2_], respectively.^2*b*^ We examined the magnetism of 2 and 5 using variable-temperature SQUID magnetometry over the temperature range 2–300 K in an external 0.5 T field, Fig. 3. For 2, the effective magnetic moment at 300 K is 2.98*μ*_B_ per U ion (4.22*μ*_B_ per complex), and this decreases only slowly until at around 50 K the *μ*_eff_ value starts to drop precipitously, reaching 1.28*μ*_B_ (1.81*μ*_B_ per complex) at 2 K. The corresponding data for 5 are quite similar, being per U ion 2.93*μ*_B_ (4.23*μ*_B_ per complex) at 300 K, again decreasingly only slowly then rapidly decreasing below 50 K reaching 1.35*μ*_B_ (1.91*μ*_B_ per complex). These data are characteristic of uranium(iv) ions that are bonded to charge-loaded, strong point donor ligands that includes oxo, imide, and nitride linkages.^15^ The magnetic data support the uranium(iv) formulations of 2 and 5, though the large low-temperature effective magnetic moments suggest that 2 and 5 do not adopt the usual magnetic singlet ground state of uranium(iv) (whose low-temperature magnetic moment would usually be essentially zero but in reality ∼0.3*μ*_B_ per U ion due to temperature-independent paramagnetism), but instead exhibit doubly degenerate ground states.^15^ This is consistent with the strong point charge nature of a nitride, and also further demonstrates that such ligands result in the effective symmetry of the crystal field that they enforce dominating over what would otherwise be the spin–orbit coupled nature of the ground multiplet.^16^

Magnetic data were modelled using the program PHI.^17^ Using the |*J* = 4, *m*_*j*_〉 basis for each ^3^H_4_ uranium(iv) ion, we modelled the magnetic data for 2 and 5 using the spin Hamiltonian (eqn (1)):1
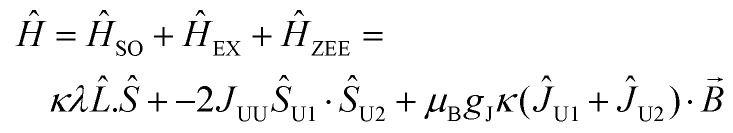
to simulate the magnetic susceptibility where *κ* is the orbital reduction factor, *λ* is the spin–orbit coupling constant (1982 cm^−1^ for U(iv)), *J* is the Lines exchange parameter, *g*_*J*_ is the Landé *g*-factor, and the exchange term is treated using a Clebsch–Gordan decomposition. The linear portions of the *χT versus T* data (100–280 K) were fit with *R*^2^ values of >0.99 yielding similar *χ*_TIP_ values of 1.3 × 10^−3^ (2) and 1.9 × 10^−3^ emu mol^−1^ (5) respectively, which were included within the model. Excellent fits were obtained, producing *J* = −4.1 cm^−1^, *κ* = 0.853, and *g* = 2.07 for 2 and *J* = −3.4 cm^−1^, *κ* = 0.822, and *g* = 1.99 for 5. We note that the *κ* values are consistent with *κ* values that have been computed for related uranium(v)–nitrides and diuranium(iv)–chalcogenide complexes,^2*a*,16^ and the *J* values indicate AF U⋯U coupling that is stronger than related U⋯U coupling in Tren^TIPS^ uranium(v)–nitrides determined by EPR,^2*a*^ similar in magnitude but of opposite sign to ferromagnetic coupling in diuranium(iv) acetylides,^18^ but towards the lower end of U⋯U AF coupling in siloxide and amide diuranium(iv)–nitride complexes that exhibit higher Néel temperatures than 2 or 5.^3*i,j*^ Indeed, the observation of U⋯U coupling remains relatively rare for uranium(iv).^3*i,j*,16,18,19^ Lastly, the *J* values for 2 and 5 are in line with their respective U⋯U distances and are in good agreement with computed values (see below).

### Computational analysis of the dinuclear nitride and nitride-imide complexes 2 and 5

In order to probe the electronic structures of 2 and 5 in greater detail, and in particular to provide insight into the nature of the U⋯U electronic communication suggested by magnetometry we examined 2 and 5 with quantum chemical computational techniques. The following discussion will largely focus on 2 for reasons that will become self-evident, but we note in passing that PBE0 hybrid DFT calculations on 5 when formulated as a diuranium(iv/v) dinitride returned U1–N6 and U1–N5 distances of 2.13 and 2.02 Å, respectively, but a diuranium(iv/iv) nitride-imide formulation produced U1–N6 and U1–N5 distances of 2.15 and 2.21 Å. The latter metrics more closely match the experimental solid-state structure than the former, and indeed are in-line with the magnetic data confirming the diuranium(iv/iv) nitride-imide formulation of 5. Quantum Theory of Atoms-in-Molecules (QTAIM)^20^ and Natural Bond Orbital (NBO)^21^ bonding metrics and charges for 5 are given in Table 1. The significantly weaker U–N_imide_ bond is reflected in both the Wiberg Bond Index (WBI) and delocalisation index *δ*(U,N_imide_) (0.84 and 0.86 respectively) *versus* that of the U–N_nitride_ bond (1.13 and 1.05 respectively).

**Table 1:** NBO and QTAIM bond indices and charges for the optimised geometry of 5 with the PBE0 functional, and the full molecule (2) and SiH_3_ model (2A), at the crystal structure geometry (XRD) and the optimised geometry (opt), with both PBE and PBE0 functionals

	Bond indices^a^						Charges^b^					
	WBI (NBO)			QTAIM *δ*(A,B)			NBO natural		Mulliken		QTAIM	
	U–N_ring_^a^	U–N_ring_^b^	U–U	U–N_ring_^a^	U–N_ring_^b^	U–U	U	N_ring_	U	N_ring_	U	N_ring_
**PBE0:**												
5-opt ^5^A_g_	1.13	0.84	0.19	1.05	0.86	0.15	1.64	−1.43, −1.30*	1.19	−0.93, −0.71*	2.30	−1.75, −1.53*
2-XRD ^5^A_g_	1.15	1.05	0.23	1.13	1.08	0.22	1.64	−1.50	1.36	-0.95	2.01	−1.68
2-opt ^5^A_g_	1.18	1.03	0.23	1.15	1.05	0.20	1.58	−1.47	1.36	-0.95	2.06	−1.68
2A-XRD ^5^A_g_	1.17	1.14	0.22	1.14	1.08	0.21	1.62	−1.47	1.51	-0.96	2.07	−1.72
2A-opt ^5^A_g_	1.23	1.14	0.28	1.21	1.13	0.27	1.32	−1.34	1.67	-0.89	1.94	−1.56
**PBE:**												
2-XRD ^5^A_g_	1.25	1.13	0.51	1.23	1.15	0.45	1.32	−1.31	1.07	-0.81	1.89	−1.58
2-opt ^5^A_g_	1.29	1.11	0.46	1.25	1.13	0.39	1.26	−1.28	1.08	-0.79	1.89	−1.57
2A-XRD ^5^A_u_	1.25	1.16	1.13	1.24	1.18	1.01	1.32	−1.30	1.21	-0.81	1.90	−1.63
2A-opt ^5^A_g_	1.32	1.22	0.57	1.29	1.19	0.53	1.09	−1.18	1.37	-0.76	1.83	−1.52

a For 5, U–N_ring_^a^ are the U–N_nitride_ bonds and U–N_ring_^b^ are the U–N_imide_ bonds (both pairs of bonds equal due to *C*_2_ symmetry).

b * is the N_imide_ charge, and for 2 U–N_ring_^a^ is the shorter pair of bonds, U–N_ring_^b^ is the longer pair (opposite pairs of bonds are equal due to *C*_*i*_ symmetry).

DFT geometry optimisations of 2 were performed using both PBE and hybrid PBE0, both on the full molecule (2-opt), and a model where isopropyl groups were replaced with hydrogens in the Tren^TIPS^ ligand (2A-opt). In addition, calculations were performed where heavy atoms were fixed at their crystal structure geometries, with only hydrogen positions optimised (2-XRD and 2A-XRD). As summarised in Table 2, the geometry optimisations on the full molecule (2-opt) are a good match for the crystal structure; PBE0 gives better agreement, with bond lengths in the U_2_N_2_ ring being within 0.02 Å of experiment, and U–N_amide_ and U–N_amine_ are within 0.05 Å. For PBE, key bond lengths are within 0.05 Å. In full geometry optimisation (with both PBE and PBE0) of the model 2A-opt, both ring U–N bonds shorten by about 0.05 Å. Also, the loss of the steric bulk of the Pr^i^ groups results in the U_2_N_2_ ring tilting, relative to the coordinating Li^+^ ions. Because of this, and to reduce computational cost, we used 2A-XRD as the geometry in our multireference calculations.

**Table 2:** Bond lengths, in ångstrom, of the U_2_N_2_ ring and its coordinating atoms, for the crystal structure (XRD) and geometry optimisations on the full molecule (2) and the model wherein Si^i^Pr_3_ groups are replaced by SiH_3_ (2A)

	U–U	U–N_ring_	U–N_amide_	U–N_amine_
2 XRD	3.367	2.148, 2.181	2.359, 2.379	2.810
PBE 2-opt	3.399	2.129, 2.185	2.331, 2.356	2.765
PBE 2A-opt	3.307	2.101, 2.143	2.316, 2.373	2.896
PBE0 2-opt	3.385	2.136, 2.189	2.327, 2.354	2.768
PBE0 2A-opt	3.323	2.110, 2.143	2.331, 2.367	2.843

**Fig. 4:**
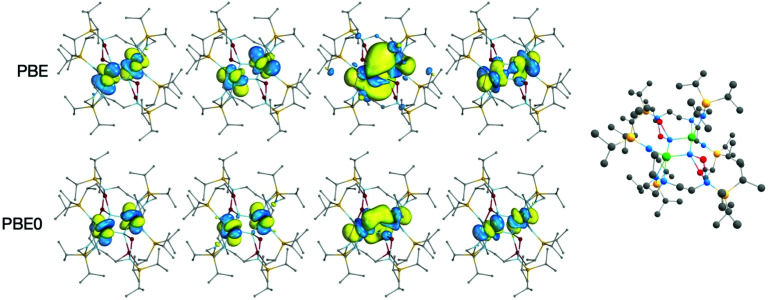
Singly-occupied α-spin Kohn Sham MOs of the ^5^A_g_ ground state of 2-opt (top: PBE, bottom PBE0). The isosurfaces enclose 90% of the orbitals. Hydrogen atoms are omitted for clarity. The ball and stick representation of 2-opt is shown to provide orientation of the molecular orbital representations.

In all DFT calculations, the ground state multiplicity was found to be a quintet, as would be anticipated from two 5f^2^ uranium(iv) ions; the singly-occupied orbitals are predominantly of 5f_U_ character, Fig. 4. Lower multiplicity single-point calculations were performed, but in some cases could not be converged (Tables S1 and S2‡) and where convergence was achieved were significantly higher in energy than the quintet ground state. The WBI and *δ*(A,B) are reasonably consistent between the two functionals in the case of the U–N_ring_ bonds, Table 1; PBE gives a 6–9% higher WBI/*δ*(U, N_ring_) *versus* PBE0. The U–N bond indices indicate a partial double bond, with one pair of bonds, U–N_ring_^a^, having slightly larger bonding metrics than the other, U–N_ring_^b^. This structure is indicative of a ring motif more like U_2_N_2_ than U_2_N_4_ studied in matrix isolation experiments. This bonding pattern is also comparable to the uranium(v) U_2_N_2_ siloxide complex obtained by Camp *et al.*,^8*a*^ which similarly features delocalised bonding in contrast to the related uranium(vi) U_2_N_2_ siloxide congener which has bonding analogous to U_2_N_4_ with pairs of triple and single bonds in the ring.^8*b*^

There is a significant difference between the PBE and PBE0 values for the U–U WBIs and *δ*(U,U), with PBE being about double that calculated for PBE0. This likely reflects the more radially extended PBE 5f_U_ orbitals shown in Fig. 4, which show increased 5f_U_ overlap. In the QTAIM calculations, there is a ring critical point at the centre of the U_2_N_2_ ring, so there is no bond critical point between the two uranium atoms. The PBE calculation on 2A-XRD is out of step with the other systems, with a U–U WBI bond index of 1.13 and *δ*(U,U) of 1.01. This is likely a result of the calculated ^5^A_u_ ground state and given the lack of consistency with other calculations is likely not reflective of the full molecule.

It is interesting to note that the U–U WBI obtained in the PBE0 calculations, 0.23 for 2-opt, is about the same as that observed in the U_2_Ni_2_ and U_2_Ni_3_ rings studied by Feng *et al.* (using the hybrid B3PW91 functional), which they suggest indicates a U–U ‘bonding interaction’.^22^ In the U_2_N_2_ ring we report, the U–U distance is 3.39 Å, *versus* around 4.3–4.5 Å for the uranium–nickel systems. The difference in U–U distance suggests that the ring geometry is driven by the U–N bonds and that any uranium–uranium interaction is weak by comparison. That the WBI is small, and virtually unchanged on shortening by around 1 Å, suggests to us there is minimal metal–metal bonding present.

The bonding description we identify for 2 – a small alternation of the U–N bonds, and, at the DFT level, a high-spin state with largely nonbonding 5f_U_ electrons – is more similar to that of U_2_N_2_ than U_2_N_4_, where distinct single and triple bonds are observed. Mindful that Vlaisavljevich *et al.* identify a highly multiconfigurational electronic structure for U_2_N_2_ at the RASPT2 level whereas a singlet, largely monoconfigurational CASSCF ground state was identified for U_2_N_4_,^9^ we performed further calculations using multiconfigurational techniques to explore whether our DFT description holds at higher levels of theory. As noted above, we performed the calculations on the 2A-XRD model system.

Our RASSCF active space follows that used by Vlaisavljevich *et al.* on molecular U_2_N_2_,^9^ considering the U_2_N_2_ core as U_2_N_2_^2+^; we include the four nonbonding 5f_U_ electrons in RAS2, the 6 σ and π U_2_N_2_ bonding orbitals in RAS1 and corresponding antibonding orbitals in RAS3, with single and double excitations allowed out of RAS1 into RAS3. To inform this choice, and especially to identify the number of nonbonding 5f_U_ orbitals to include in RAS2, we first performed preliminary CASSCF and CASPT2 calculations, including only 5f_U_ orbitals in our active space. Given the ^3^H uranium(iv) ground state level, eleven low-lying states would be anticipated.^23^ To see if this the case, we performed a 20 state average (SA) ^5^A_g_ (the DFT ground state) [4,14] CASPT2 calculation. We observe (Table S3‡) a small (0.05 eV) jump at the 12^th^ state, as expected; however, an 11 state calculation would necessitate 12 5f_U_ orbitals in the active space, which proved too large when including the U_2_N_2_ bonding and antibonding orbitals. We therefore chose to focus on a 5 state average, including the ground state and other near-degenerate states while including 10 5f_U_ orbitals; the difference in the [4,10] and [4,14] CASSCF energies is 0.05 eV for 5 states, rising to 0.09 eV for 6 and 0.20 eV for 11 states (Table S4‡). Our active space for our RASSCF and RASPT2 calculation then corresponds to (16,2,2;6,10,6) in the Sauri notation.

We performed 5-SA RASSCF calculations for singlet, triplet and quintet spin multiplicities in A_g_ and A_u_ symmetries, and MS-RASPT2 calculations on these references. The relative MS-RASPT2 energies are given in Table 3, and relative and absolute MS-RASPT2 and SA-RASSCF energies given in Tables S5 and S6‡ respectively. There are 9 states within 0.03 eV and 18 states within 0.1 eV. These states differ only in the occupation of the nonbonding 5f_U_ orbitals in RAS2; the occupation of the bonding orbitals in RAS1 and antibonding orbitals in RAS3 is essentially identical in each state, meaning that the U_2_N_2_ ring bonding is the same (Table S7‡). Note that the effects of spin–orbit coupling have been neglected. Calculation of enough excited states to perform a RAS State Interaction (RASSI) would likely be challenging, given the large number of low-lying states identified in this study, and also that Vlaisavljevich *et al.* were unable to calculate enough states to perform such a calculation on the bare U_2_N_2_ molecule. Since all states identified have very similar qualitative electronic structures, our conclusions would very likely be unaltered at the RASSI level.

**Table 3:** The relative energies of the MS-RASPT2 calculations on 2A-XRD, for each space symmetry and spin multiplicity, in eV. The ground state is ^1^A_g_

State:	^1^A_g_	^1^A_u_	^3^A_g_	^3^A_u_	^5^A_g_	^5^A_u_
1	**0.000**	0.011	0.020	0.009	0.002	0.010
2	0.018	0.078	0.089	0.027	0.022	0.082
3	0.060	0.086	0.092	0.067	0.061	0.084
4	0.103	0.154	0.161	0.110	0.103	0.150
5	0.145	0.225	0.234	0.154	0.145	0.226

The change in ground state multiplicity, ^1^A_g_ at the MS-RASPT2 level *vs.*^5^A_g_ with DFT, suggests weak AF coupling between the two uranium(iv) centres in 2; the 1.7 meV difference between the lowest energy ^1^A_g_ and ^5^A_g_ MS-RASPT2 states corresponds to an exchange coupling parameter of −7.0 cm^−1^, which is in good agreement with the value of −4.1 cm^−1^ obtained from the spin Hamiltonian modelling of the magnetic data. Consistent with the suggested weak AF coupling, a Weiss constant of −8 K is computed, which compares very well with the experimental value of −7.95 K. Overall, the excellent agreement between experimental and computed magnetic properties of 2 underscores the validity of our computational model.

**Fig. 5:**
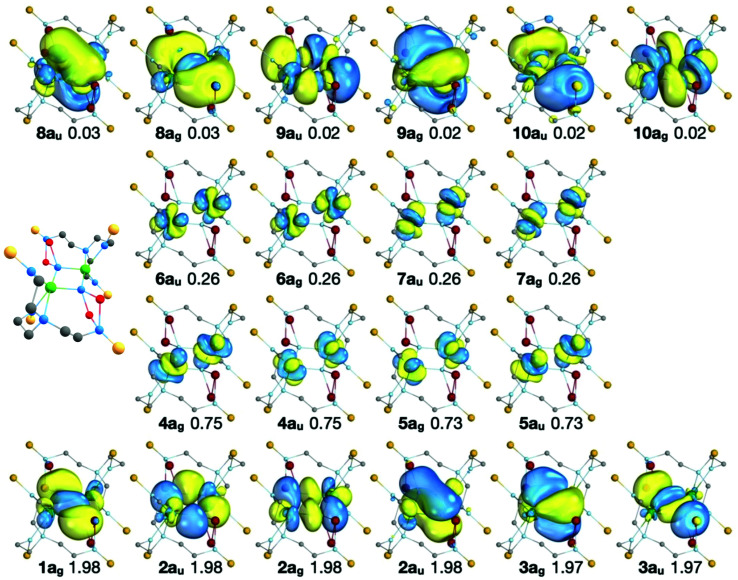
The natural orbitals, their symmetries, and occupation numbers of the SA-RASSCF state which most contributes (66.3%) to the ^1^A_g_ MS-RASPT2 ground state of 2A. Orbitals with occupancies >0.01 are shown. The isosurfaces enclose 90% of the orbitals. RAS1: bottom row, RAS2: middle rows, RAS3: top row. Note that the natural orbitals of the other states which contribute to the ^1^A_g_ MS-RASPT2 ground state are very similar to those shown here, differing only in the occupation of the RAS2 orbitals (see Table S7 of the ESI‡). The ball and stick representation of 2A is shown to provide orientation of the molecular orbital representations.

The RASSCF active natural orbitals of the state which most contributes (66.3%) to the ^1^A_g_ MS-RASPT2 ground state are shown in Fig. 5. The natural orbitals do not suggest any significant direct U–U bond; in and out-of-phase linear combinations of 5f_U_ orbitals are almost exactly equally occupied, and bonding orbitals in RAS1 are dominated by nitride contributions.

**Fig. 6:**
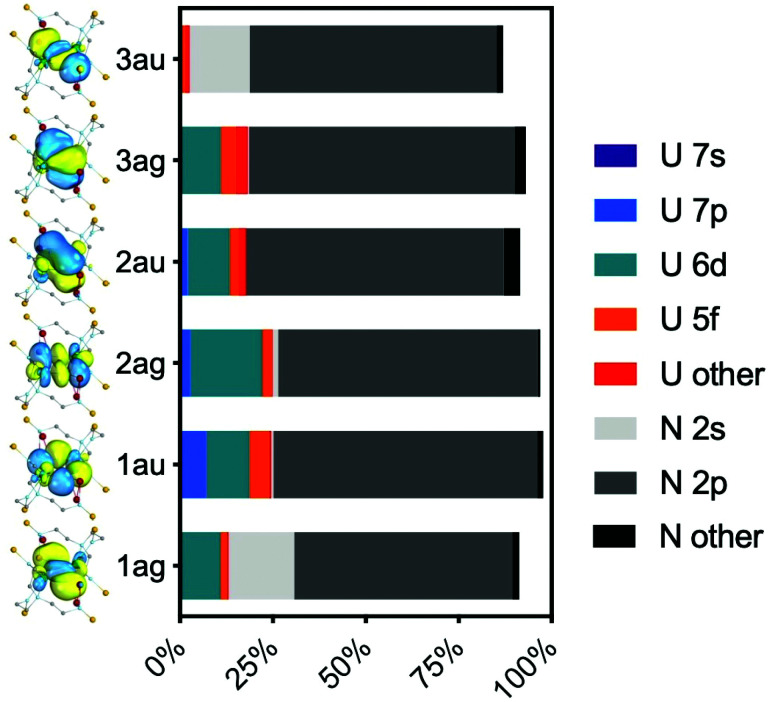
Mulliken analysis of the RAS1 bonding orbitals shown in Fig. 5.

The active orbitals are highly localised on the U_2_N_2_ ring; the bonding orbitals (RAS1) are at least 90% localised on the U_2_N_2_ ring. Population analysis of the composition of the bonding orbitals (Fig. 6 and Table S8‡) highlights the larger contributions of the 6d orbitals compared with the 5f. The remaining orbitals are similarly highly localised; the nonbonding RAS2 orbitals are at least 94% 5f_U_ (Table S9‡) and antibonding RAS3 orbitals at least 85% localised on the U_2_N_2_ ring (Table S10‡).

The character of the six ring bonding orbitals is similar to that of U_2_N_2_, featuring 4 delocalised σ bonds and 2 delocalised π bonds, and agrees with the qualitative bonding description provided by Vlaisavljevich *et al.*^9^ For 2A-XRD, the average U–N effective bond order (EBO) in the ring is 1.47. However, there is only a small population on uranium of 3.5% for orbital 3a_u_ (Fig. 6), and the corresponding antibonding orbital in RAS3, 10a_u_, of 8.9% (Table S10‡). Sharma *et al.* suggested an EBO threshold of 10% uranium population in their recent study of uranium-transition metal complexes;^24^ given this, it may therefore be more appropriate to classify these orbitals as nonbonding in which case we arrive at an EBO of 1.22. The overall conclusion is hence that 2 is a very unusual case where PBE analysis suggests the presence of uranium–uranium bonding, but moving to the PBE0 functional, which should produce more localised 5f orbitals, yields a different picture. The latter is supported by high-level *ab initio* calculations; whilst there could be some uranium–uranium bonding in 2, it is all but cancelled out by almost equal populations of bonding and anti-bonding orbital combinations, and is at best a very minor component of bonding that is dominated by uranium–nitride bonding. The uranium–uranium AF coupling evidenced by experimental magnetometry and reproduced in the calculations is thus primarily the result of nitride mediated super-exchange and not metal–metal bonding.

## Conclusions

To conclude, we have examined the reduction of 1 past the initial nitride formation step, to isolate doubly reduced 2, where stabilisation of the uranium–nitride linkage is seemingly facilitated by the small and highly polarising Li ions; in contrast, heavier alkali metals accomplish mono-reduction or over-reduction. The reduction sequence has been examined, revealing insight into the reduction sequence 1 → 4 → 2 → 3. Attempts to isolate odd-electron species resulted in exchange of Li for H, converting dinitride 2 to nitride-imide 5. Whilst terminal uranium(v/vi)–nitrides have been accessed using the Tren^TIPS^ ancillary ligand, our attempts to access a terminal uranium(iv)–nitride here have resulted in the dimeric species 2, highlighting that charge build-up at the nitride is stabilised by oligomerisation. This highlights that uranium(iv) is not such a favourable oxidation state to be paired with a nitride compared to uranium(v/vi), which is consistent with hard–soft acid–base expectations. The ‘diamond-core’ structural motif of tetravalent 2 is notable, because this motif is usually found with higher oxidation state uranium(v/vi)–nitrides or tetrauranium(iv) clusters, with mono-nitrides dominating the landscape of diuranium(iv)–nitrides.

The variable-temperature magnetic data for 2 and 5 reveal another two instances of high low-temperature magnetic moments for uranium(iv), likely the result of a doubly, not singly, degenerate ground states, where the strong crystal field of the nitride presents an effective symmetry that dominates over the otherwise spin–orbit coupled nature of the ground multiplet of uranium(iv). The magnetic data for 2 and 5 also reveal still relatively rare instances of AF U⋯U coupling for uranium(iv).

In order to understand the nature of the U⋯U electronic coupling in 2, we have deployed single- and multi-reference computational methods which accurately reproduce the magnetic data. Through this approach, we have identified an unusual borderline case, which initially raised the prospect of direct uranium–uranium bonding, but in-depth computational analysis reveals that if any uranium–uranium bonding is present it is rather weak, and instead the nitride centres dominate the mediation of the electronic communication between the uranium(iv) ions. Our computational results thus emphasise the importance of obtaining high-level *ab initio* insight when probing potential actinide-actinide electronic communication and bonding in weakly coupled systems. Lastly, this computational analysis highlights analogies between the ‘diamond-core’ dinitride of 2 reported here and matrix-isolated binary U_2_N_2_, potentially providing conceptual links between microscopic inert matrix and isolable macroscopic species.

## Author contributions

D.M.K., M.G., and L.C. prepared and characterised the compounds. B.E.A. and N.K. carried out and analysed the quantum chemical calculations. J.A.S. acquired and analysed the magnetic data. A.J.W. collected, solved, and refined the crystal structures. S.T.L. conceived and directed the research, analysed all the data, and wrote the manuscript with input from all authors.

## Data availability

Data can be found in the ESI‡ or are available from the authors on request.

## Conflicts of interest

There are no conflicts to declare.

## Supplementary Material

DT-051-D2DT00998F-s001

DT-051-D2DT00998F-s002
