# Supplementary material for: Uranium–nitride chemistry: uranium–uranium electronic communication mediated by nitride bridges
Source: Dalton Trans. 2022 May 20;51(22):8855–64. doi: 10.1039/d2dt00998f (PMC9171730; doi:10.1039/d2dt00998f)
Supplement: DT-051-D2DT00998F-s001 [file DT-051-D2DT00998F-s001.pdf]

Electronic Supplementary Material (ESI) for Dalton Transactions.
This journal is © The Royal Society of Chemistry 2022

- Electronic Supplementary Information -

Uranium-Nitride Chemistry: Uranium-Uranium Electronic Communication Mediated by Nitride Bridges

David M. King, Benjamin E. Atkinson, Matthew Gregson, Lucile Chatelain, John A. Seed, Ashley J. Wooles, Nikolas Kaltsoyannis,* and Stephen T. Liddle*

Department of Chemistry, University of Manchester, Oxford Road, Manchester, M13 9PL, UK.

*Email: steve.liddle@manchester.ac.uk; nikolas.kaltsoyannis@manchester.ac.uk

Experimental Details

General

All manipulations were carried out under an inert atmosphere of dry nitrogen using Schlenk techniques, or in an MBraun UniLab glovebox operating under an atmosphere of dry nitrogen with H_2O and O_2 < 0.1 ppm. All glassware was silylated and dried either by overnight storage in an oven at 200 °C or by flame-drying under 10^{-3} mm Hg vacuum. Solvents were dried by passage through activated alumina towers and degassed prior to use. All solvents were stored over potassium mirrors, except for ethers that were stored over activated 4Å molecular sieves. Deuterated solvent was distilled from NaK_2, degassed by three freeze-pump-thaw cycles and stored under nitrogen.

Crystals were examined using an Agilent Supernova diffractometer, equipped with an Atlas CCD area detector and mirror-monochromated Cu Kα radiation (λ = 1.5418 Å). Intensities were integrated from a sphere of data recorded on narrow (1.0°) frames by ω rotation. Cell parameters were refined from

the observed positions of all strong reflections in each data set. Gaussian grid face-indexed absorption corrections with a beam profile correction were applied.[1] The structures were solved by direct methods using ShelXS,[2] and all non-hydrogen atoms were refined by full-matrix least-squares on all unique F^2 values with anisotropic displacement parameters with exceptions noted in the respective cif files. Except where noted, Hydrogen atoms were refined with constrained geometries and riding thermal parameters. The *N*-H atom in **5** was located in the Fourier difference map. CrysAlisPro[3] was used for control and integration, and SHELXL[4] and Olex2[5] were employed for structure refinement. ORTEP-3[6] and POV-Ray[7] were employed for molecular graphics. FTIR spectra were recorded on a Bruker Alpha spectrometer with a Platinum-ATR module in the glovebox. Static variable-temperature magnetic moment data were recorded in an applied DC field of 0.5 T on a Quantum Design MPMS3 XL7 superconducting quantum interference device (SQUID) using recrystallised powdered samples. Samples were carefully checked for purity and data reproducibility between independently prepared batches for each compound examined. Care was taken to ensure complete thermalisation of the sample before each data point was measured, and samples were immobilised in an eicosane matrix to prevent sample reorientation during measurements. Diamagnetic corrections were applied using tabulated Pascal constants and measurements were corrected for the effect of the blank sample holders (flame sealed Wilmad NMR tube and straw) and eicosane matrix. Elemental microanalyses were carried out by Mr Martin Jennings at the Micro Analytical Laboratory, School of Chemistry, University of Manchester.

The compounds [$(\mathrm{Tren}^{\mathrm{TIPS}})\mathrm{U}(\mathrm{N}_3)$] (**1**) and [$\{(\mathrm{Tren}^{\mathrm{TIPS}})\mathrm{UNLi}\}_2$] (**4**) were prepared as described previously.[8,9] Alkali metals were washed with hexane to remove any protective mineral oil coatings and were stored under argon. The lithium powder was then used directly but other alkali metals were freshly cut and freed of any passivated oxide layer before use.

Preparation of [{(Tren^{TIPS})UN}$_2$Li$_4$] (2)

Method A: A solution of **1** (3.57 g, 4.00 mmol) in toluene (10 ml) was added to a cold (−78 °C) slurry of Li metal (0.20 g, 28.57 mmol) in toluene (20 ml). The mixture was allowed to slowly warm to room temperature and was then stirred for 5 days. Each day the mixture was sonicated for 1 hr. After this time the mixture turned dark blue/red and a red precipitate had formed. The red precipitate was isolated by filtration (via cannula), then extracted into boiling toluene (60 ml) and filtered through a frit. The residue was washed with boiling toluene (2 × 10 ml) and filtered. The combined filtrate was concentrated to ~30 ml and stored at −30 °C to yield **2** as a red crystalline solid. The product was isolated by filtration, washed with pentane (2 x 10 ml) and dried *in vacuo*. *Method B*: A solution of **4** (3.48 g, 2.00 mmol) in toluene (10 ml) was added to a cold (−78 °C) slurry of Li metal (0.04 g, 5.8 mmol) in toluene (20 ml). The mixture was allowed to slowly to warm to room temperature and was then stirred for 5 days. Each day the mixture was sonicated for 1 hr. The resulting red precipitate was extracted into boiling toluene (60 ml) and filtered through a frit. The residue was washed with boiling toluene (2 x 10 ml) and filtered. The combined filtrate was stored at −30 °C to yield **2** as a red crystalline solid. The product was isolated by filtration, washed with pentane (2 × 10 ml) and dried *in vacuo*. Representative yield of either method: 2.05 g, 58%. Anal. calcd for $C_{66}H_{150}N_{10}Li_4Si_6U_2$: C, 45.14; H, 8.61; N, 7.97%. Found: C, 45.45; H, 8.57; N, 7.88%. FTIR ν/cm^{-1} (Nujol): 1631 (w), 1377 (w), 1300 (w), 1261 (w), 1052 (bs), 1025 (s), 990 (w), 933 (s), 917(m), 882 (s), 738 (s), 671 (m), 620 (m), 564 (w), 513 (w). Once obtained in crystalline form, **2** is insoluble in aromatic solvents and it decomposes in polar solvents so ^{1}H and ^{29}Si NMR and UV/Vis/NIR spectra could not be obtained.

Preparation of [{(Tren^{TIPS})UN}$_2$HLi$_3$] (5)

Method A: Toluene (15 ml) was added to a mixture of **2** (0.44 g, 0.25 mmol) and benzo-9-crown-3 (0.18 g, 1 mmol). The resulting red mixture was gently heated to dissolve both reagents, then filtered and the volume was reduced to ca. 5 ml. Storage of the mixture at −30 °C afforded red crystals of **5**. Yield: 0.06 g, 13%. *Method B*: Toluene (10 ml) was added to a pre-cooled (−78 °C) mixture of **2** (0.20

g, 0.11 mmol) and $AgBPh_4$ (0.048 g, 0.11 mmol). The resulting red suspension was allowed to warm to room temperature, sonicated for 1 hr, then stirred for 72 hrs. Volatiles were removed *in vacuo* and the resulting red solid was recrystallised from hot toluene (2 ml) to afford red crystals of **5** on storing at room temperature. Yield: 0.11 g, 53%. Anal. Calcd for $C_{66}H_{151}Li_3N_{10}Si_6U_2$: C, 45.29; H, 8.69; N, 8.00%. Found: C, 45.34; H, 8.67; N, 7.88%. FTIR ν/cm^{-1} (ATR): 3395 (br), 2937 (m), 2855 (s), 2830 (m), 1494 (w), 1456 (m), 1241 (s), 1185 (w), 1074 (m), 1065 (m), 988 (s), 915 (s), 881 (s), 816 (m), 778 (m), 737 (s), 670 (m), 572 (s), 514 (w), 493 (m), 414(w). Once obtained in crystalline form, **5** is insoluble in aromatic solvents and it decomposes in polar solvents so ^{1}H and ^{29}Si NMR and UV/Vis/NIR spectra could not be obtained.

Representative attempted double reductions of 1 with excess Na-Cs or MC_8 (M = K-Cs)

In a typical procedure, toluene (20 ml) was added to a precooled (−78 °C) mixture of **1** (0.45 g, 0.50 mmol) and the respective reductant (2.00 mmol). The resulting mixture was allowed to warm to ambient temperature and stirred for 24 hrs, during which time it was sonicated three times (1 hr each time) before being stirred for another 24 hrs. Hot filtration then cooling afforded crystalline **4M** (typically in ~55% yield). Extended reaction times resulted in overall decomposition to Tren^{TIPS}H_3 and other unidentified species.

Representative attempted double reductions of 1 with stoichiometric Na-Cs or MC_8 (M = K-Cs)

In a typical procedure, toluene (20 ml) was added to a precooled (−78 °C) mixture of **1** (0.45 g, 0.50 mmol) and the respective reductant (1.00 mmol). The resulting mixture was allowed to warm to ambient temperature and stirred for 24 hrs, during which time it was sonicated three times (1 hr each time) before being stirred for another 24 hrs. Hot filtration then cooling afforded crystalline **4M** (typically in 45% yield). Extended reaction times resulted in overall decomposition to Tren^{TIPS}H_3 and other unidentified species.

Representative attempted reductions of 4M (M = Na-Cs) using excess Na-Cs or MC_8 (M = K-Cs)

In a typical procedure, toluene (20 ml) was added to a precooled (−78 °C) mixture of **4M** (0.50 mmol) and the respective reductant (2.00 mmol). The resulting mixture was allowed to warm to ambient temperature and stirred for 24 hrs, during which time it was sonicated three times (1 hr each time) before being stirred for another 24 hrs. Hot filtration then cooling afforded crystalline 4M (typically in 40% yield). Extended reaction times resulted in overall decomposition to $Tren^{TIPS}H_3$ and other unidentified species.

Computational Details

DFT calculations on **4** were performed with Gaussian 16 revision A.03,[10] and with Turbomole 7.3 for **3**.[11] Calculations were spin unrestricted and used the GGA functional PBE,[12] as well as the hybrid PBE0.[13] The 60e$^-$ relativistic effective core potential (RECP) of the Stuttgart/Cologne group (ECP60MWB) was used alongside the associated segmented basis set,[14-16] and on other elements the cc-pVDZ basis set was used.[17-19] Grimme's D3 dampening function was used to account for dispersion interactions.[20] Integration grids and convergence criteria were left at their default in Gaussian 16, and in Turbomole the m4 integration grid was used, with convergence criteria being left at their default. CASSCF and RASSCF calculations were performed on model systems using OpenMolcas 18.09[21] Calculations were performed in C_i symmetry, reflecting the symmetry of the XRD crystal structure. The ANO-RCC basis set was used; on uranium, and the ring nitrogen atoms, the VTZP contraction was used and VDZ on all other atoms. The second-order Douglas-Kroll-Hess Hamiltonian was used to account for scalar relativistic effects. Cholesky decomposition was used, with the high decomposition threshold. CASPT2 and MS-CASPT2 calculations used an imaginary shift of 0.2 in addition to the default IPEA shift of 0.25. Mulliken composition of the active natural orbitals was analysed with Molpy.[22] Quantum Theory of Atoms-in-Molecules (QTAIM)[23] analyses were performed with AIMALL,[24] Natural Bond Orbital (NBO) analyses were performed with NBO 6.0.[25]

Magnetic Data

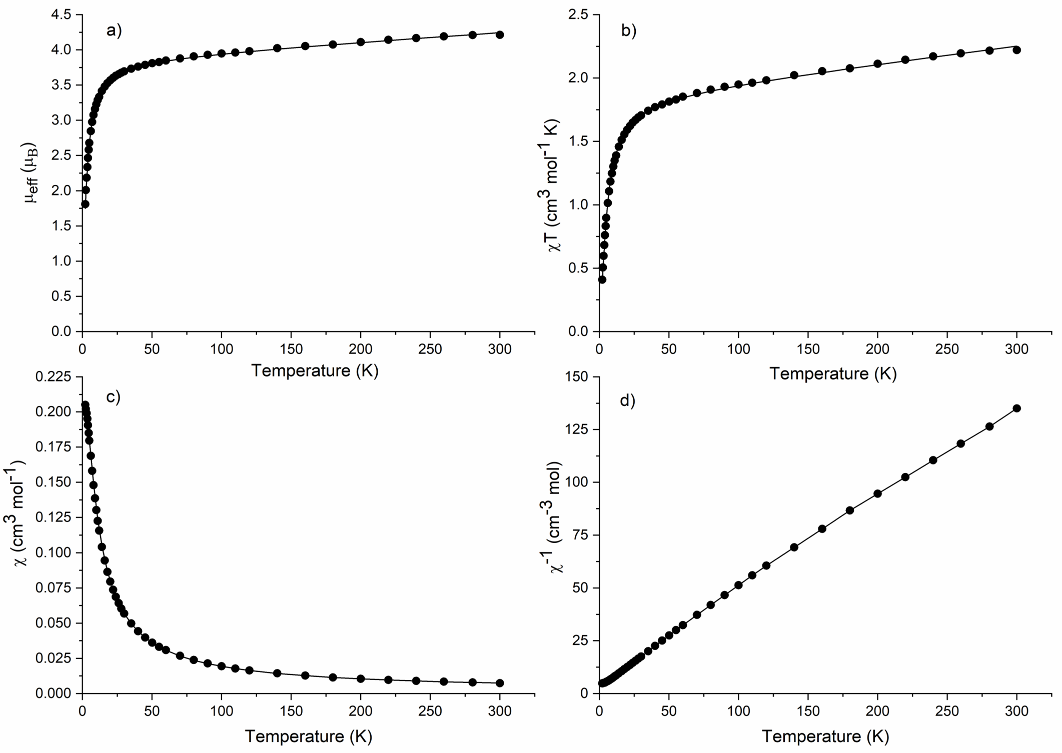

Figure S1. Variable-temperature magnetic data of a powdered sample of **2**. a) μ_{eff} vs T, b) χT vs T, c) χ vs T, d) χ^{-1} vs T. The lines represent the modelled magnetic susceptibility from PHI.

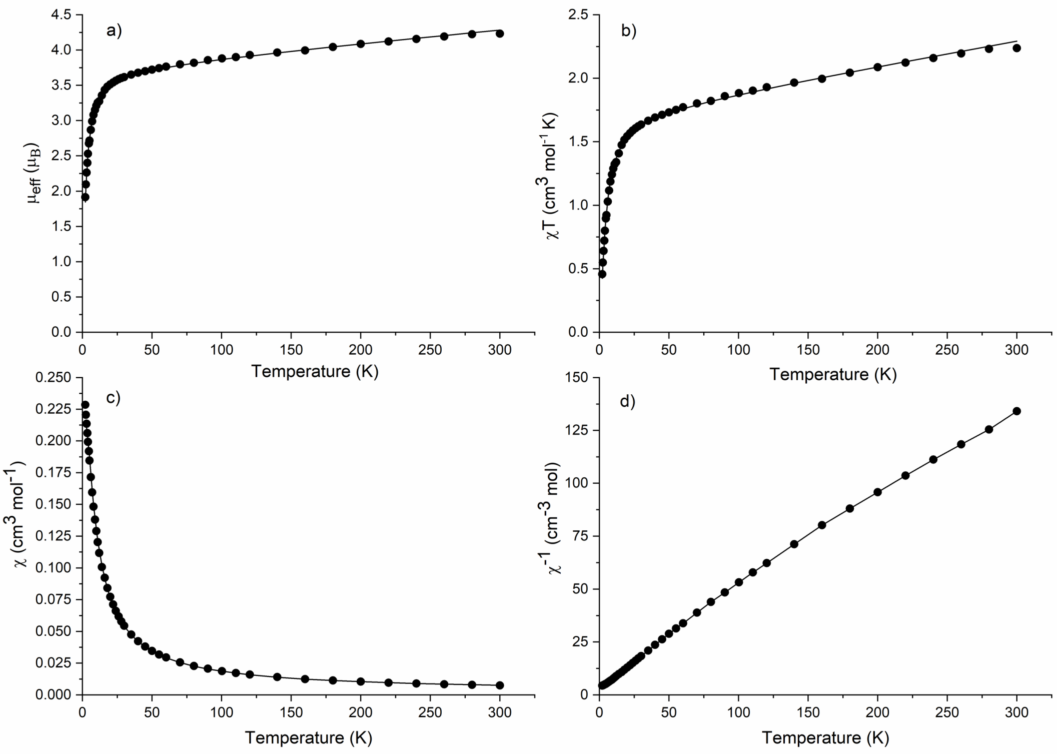

Figure S2. Variable-temperature magnetic data of a powdered sample of **5**. a) μ_{eff} vs T, b) χT vs T, c) χ vs T, d) χ^{-1} vs T. The lines represent the modelled magnetic susceptibility from PHI.

Computational Tables

Table S1. Absolute and relative energies of **2** and **2A** with the functional PBE, for several multiplicities at the quintet optimised geometry. * = failed to converge.

	**2**-opt		**2**-XRD		**2A**-opt		**2A**-XRD	
2S+1	E / Ha	ΔE / eV	E / Ha	ΔE / eV	E / Ha	ΔE / eV	E / Ha	ΔE / eV
1	*	*	*	*	-3749.25622	1.233	*	*
3	-5869.45155	0.483	-5869.44815	0.360	-3749.28408	0.475	-3749.22947	0.535
5	-5869.46929	0.000	-5869.46136	0.000	-3749.30153	0.000	-3749.24913	0.000

Table S2. Absolute and relative energies of **2** and **2A** with the functional PBE, for several multiplicities at the quintet optimised geometry. * = failed to converge.

	**2**-opt		**2**-XRD		**2A**-opt		**2A**-XRD	
2S+1	E / Ha	ΔE / eV	E / Ha	ΔE / eV	E / Ha	ΔE / eV	E / Ha	ΔE / eV
1	-5870.08694	2.708	*	*	-3749.42771	2.249	-3749.36165	2.695
3	-5870.17328	0.358	-5870.15591	0.451	-3749.44555	1.763	-3749.39699	1.733
5	-5870.18644	0.000	-5870.17248	0.000	-3749.51035	0.000	-3749.46067	0.000

Table S3. Absolute and relative energies CASPT2 energies of **2A**-XRD (^5A_g, 20-SA [4,14] CASSCF reference).

State	E / Ha	ΔE / eV
1	-58695.48117	0.00
2	-58695.48111	0.00
3	-58695.47995	0.03
4	-58695.47988	0.04
5	-58695.47985	0.04
6	-58695.47975	0.04
7	-58695.47916	0.05
8	-58695.47904	0.06
9	-58695.47870	0.07
10	-58695.47817	0.08
11	-58695.47785	0.09
12	-58695.47607	0.14
13	-58695.47423	0.19
14	-58695.47420	0.19
15	-58695.47409	0.19
16	-58695.47364	0.20
17	-58695.47300	0.22
18	-58695.47270	0.23
19	-58695.47253	0.24
20	-58695.47230	0.24

Table S4. The CASSCF absolute energies in Hartree with a [4,14] and [4,10] active space, and the difference in energy between the two active spaces, in eV. Absolute energies are shifted up by 58690 Hartree. Energies shown for a 20, 11, 6 and 5 state average on **2A**-XRD (^5A_g).

.	20 SA			11 SA			6 SA			5 SA		
State:	E[4,14] Ha	/E[4,10] Ha	/ΔE / eV	E[4,14] Ha	/E[4,10] Ha	/ΔE / eV	E[4,14] Ha	/E[4,10] Ha	/ΔE / eV	E[4,14] Ha	/E[4,10] Ha	/ΔE / eV
1	-0.53102	-0.53120	-0.01	-0.53339	-0.53222	0.03	-0.53427	-0.53367	0.02	-0.53453	-0.53403	0.01
2	-0.52976	-0.52953	0.01	-0.53209	-0.53053	0.04	-0.53249	-0.53193	0.02	-0.53266	-0.53209	0.02
3	-0.52857	-0.52796	0.02	-0.53086	-0.52893	0.05	-0.53079	-0.53027	0.01	-0.53087	-0.53023	0.02
4	-0.52688	-0.52593	0.03	-0.52702	-0.52627	0.02	-0.52788	-0.52587	0.05	-0.52779	-0.52590	0.05
5	-0.52562	-0.52432	0.04	-0.52572	-0.52465	0.03	-0.52611	-0.52417	0.05	-0.52593	-0.52399	0.05
6	-0.52545	-0.52231	0.09	-0.52558	-0.52268	0.08	-0.52584	-0.52252	0.09			
7	-0.52496	-0.52072	0.12	-0.52495	-0.52104	0.11						
8	-0.52459	-0.52067	0.11	-0.52456	-0.52038	0.11						
9	-0.52422	-0.51798	0.17	-0.52429	-0.51714	0.19						
10	-0.52375	-0.51702	0.18	-0.52373	-0.51673	0.19						
11	-0.52318	-0.51630	0.19	-0.52309	-0.51546	0.21						
12	-0.52280	-0.51339	0.26									
13	-0.52134	-0.51326	0.22									
14	-0.52095	-0.51286	0.22									
15	-0.52038	-0.51166	0.24									

16	-0.51982	-0.51068	0.25
17	-0.51945	-0.50913	0.28
18	-0.51901	-0.50906	0.27
19	-0.51888	-0.50812	0.29
20	-0.51849	-0.50547	0.35

Table S5. The absolute (Ha) and relative (eV) energies of the MS-RASPT2 calculations on **2A**-XRD, for each space symmetry and spin multiplicity. Absolute energies are shifted up by 58695 Hartree.

State:	^1A_g		^1A_u		^3A_g		^3A_u		^5A_g		^5A_u	
	E / Ha	ΔE / eV	E / Ha	ΔE / eV	E / Ha	ΔE / eV	E / Ha	ΔE / eV	E / Ha	ΔE / eV	E / Ha	ΔE / eV
1	-0.4528	0.0000	-0.4524	0.0112	-0.4521	0.0196	-0.4524	0.0090	-0.4527	0.0017	-0.4524	0.0103
2	-0.4521	0.0179	-0.4499	0.0777	-0.4495	0.0889	-0.4518	0.0272	-0.4520	0.0216	-0.4498	0.0816
3	-0.4506	0.0595	-0.4496	0.0855	-0.4494	0.0922	-0.4503	0.0672	-0.4505	0.0609	-0.4497	0.0840
4	-0.4490	0.1029	-0.4471	0.1542	-0.4469	0.1609	-0.4487	0.1096	-0.4490	0.1028	-0.4472	0.1504
5	-0.4475	0.1446	-0.4445	0.2254	-0.4442	0.2341	-0.4471	0.1536	-0.4474	0.1452	-0.4445	0.2257

Table S6. The absolute (Ha) and relative (eV) energies of the SA-RASSCF calculations on **2A**-XRD, for each space symmetry and spin multiplicity. Absolute energies are shifted up by 58695 Hartree.

State:	^1A_g		^1A_u		^3A_g		^3A_u		^5A_g		^5A_u	
	E / Ha	ΔE / eV	E / Ha	ΔE / eV	E / Ha	ΔE / eV	E / Ha	ΔE / eV	E / Ha	ΔE / eV	E / Ha	ΔE / eV
1	-0.6944	0.0084	-0.6913	0.0929	-0.6914	0.0897	-0.6945	0.0058	-0.6947	0.0000	-0.6917	0.0827
2	-0.6925	0.0595	-0.6860	0.2366	-0.6861	0.2337	-0.6926	0.0576	-0.6927	0.0538	-0.6863	0.2272
3	-0.6906	0.1122	-0.6849	0.2677	-0.6850	0.2648	-0.6907	0.1095	-0.6909	0.1042	-0.6852	0.2586
4	-0.6863	0.2284	-0.6839	0.2937	-0.6840	0.2921	-0.6864	0.2262	-0.6866	0.2214	-0.6841	0.2888
5	-0.6844	0.2811	-0.6828	0.3248	-0.6828	0.3229	-0.6845	0.2786	-0.6846	0.2734	-0.6829	0.3197

Table S7. The occupation numbers of the natural orbitals for each root of the ^1A_g SA-RASSCF calculation on **2A**-XRD.

Root:	Orbital symmetry:	RAS1			RAS2					RAS3		
1	a_g	1.974941	1.978228	1.979562	0.132297	0.864374	0.000280	0.883054	0.132114	0.019071	0.022107	0.024979
	a_u	1.977365	1.974681	1.979150	0.872186	0.131708	0.000241	0.132193	0.853635	0.019788	0.025194	0.022852
2	a_g	1.974960	1.978255	1.979568	0.426622	0.561836	0.006958	0.815791	0.197892	0.019088	0.022378	0.024992
	a_u	1.977365	1.974684	1.979147	0.568341	0.430304	0.006855	0.196098	0.790811	0.019934	0.025247	0.022875
3	a_g	1.974989	1.978292	1.979576	0.734487	0.258218	0.000317	0.751834	0.261021	0.019088	0.022308	0.025000
	a_u	1.977371	1.974688	1.979155	0.262105	0.745485	0.000275	0.259551	0.728330	0.019864	0.025176	0.022871
4	a_g	1.974965	1.978238	1.979576	0.065760	0.835375	0.153852	0.536700	0.414465	0.019023	0.021872	0.024972
	a_u	1.977399	1.974661	1.979094	0.842425	0.065037	0.410875	0.155042	0.523146	0.019679	0.025005	0.022838
5	a_g	1.974986	1.978264	1.979582	0.515704	0.535886	0.146921	0.469114	0.334837	0.019039	0.021999	0.024994
	a_u	1.977403	1.974662	1.979096	0.550010	0.515886	0.145323	0.331305	0.457414	0.019717	0.025006	0.022851

Table S8. Composition analysis of the RAS1 active orbitals of the SA-RASSCF state which most contributes (66.3%) to the $^{1}A_g$ MS-RASPT2 ground state of **2A**.

	$1a_g$	$1a_u$	$2a_g$	$2a_u$	$3a_g$	$3a_u$
U total	13.066	24.546	24.934	17.837	18.251	2.609
U 7s	-1.196	7.145	-0.236	-0.008	0.161	0.087
U 6p	0.222	-0.071	3.068	2.062	0.032	-1.609
U 6d	11.940	11.687	19.308	11.361	10.858	-0.640
U 5f	1.764	5.060	2.583	2.286	3.914	3.526
N total	78.259	73.173	72.024	73.686	74.777	84.323
N 2s	17.663	0.644	1.491	0.011	0.406	16.209
N 2p	58.656	70.961	69.799	69.265	71.412	66.406
U + N	91.325	97.719	96.958	91.522	93.028	86.932
U / (N + U)	14.307	25.119	25.716	19.489	19.619	3.002
Occupation	1.980	1.979	1.978	1.977	1.975	1.975

Table S9. Composition analysis of the RAS2 active orbitals of the SA-RASSCF state which most contributes (66.3%) to the $^{1}A_g$ MS-RASPT2 ground state of **2A**.

	$4a_g$	$4a_u$	$5a_g$	$5a_u$	$6a_g$	$6a_u$	$7a_g$	$7a_u$
U total	99.448	99.512	99.354	99.443	99.069	98.307	98.323	99.449
U 7s	2.072	0.057	0.133	1.466	0.244	0.351	0.320	0.167
U 6p	0.077	0.065	0.004	-0.091	-0.042	-0.001	0.135	0.197
U 6d	2.200	0.766	1.217	1.069	1.241	0.272	1.231	0.375
U 5f	94.805	98.241	97.583	96.518	97.354	96.881	95.816	98.294
N total	0.386	0.158	0.314	0.165	0.354	0.712	0.696	0.104
N 2s	-0.007	-0.008	0.000	-0.002	0.000	0.017	0.000	0.009
N 2p	0.226	0.108	0.270	0.046	0.240	0.558	0.481	0.022
U + N	99.834	99.670	99.668	99.608	99.423	99.019	99.019	99.553
U / (N + U)	99.613	99.842	99.685	99.835	99.644	99.281	99.297	99.896
Occupation	0.752	0.746	0.735	0.728	0.262	0.261	0.260	0.258

Table S10. Composition analysis of the RAS2 active orbitals of the SA-RASSCF state which most contributes (66.3%) to the $^{1}A_g$ MS-RASPT2 ground state of **2A**.

	$8a_g$	$8a_u$	$9a_g$	$9a_u$	$10a_g$	$10a_u$
U total	19.019	18.865	26.844	22.746	8.928	34.130
U 7s	0.001	0.691	13.053	0.245	1.091	0.071
U 6p	1.914	0.951	-4.281	0.048	1.326	3.551
U 6d	15.673	15.843	15.469	18.118	0.929	29.930
U 5f	0.927	1.860	4.079	2.234	4.843	2.747
N total	71.791	70.982	67.508	69.352	75.934	63.689
N 2s	0.054	1.322	0.057	-0.000	1.355	0.027
N 2p	23.992	15.666	20.232	21.040	9.090	19.540
U + N	90.809	89.847	94.352	92.098	84.862	97.818
U / (N + U)	20.943	20.997	28.451	24.697	10.521	34.891
Occupation	0.025	0.025	0.023	0.022	0.020	0.019

References

1. P. Coppens, W.C. Hamilton, *Acta Cryst. Sect A*, 1970, **A26**, 71-83.

2. G.M. Sheldrick, *Acta Cryst. Sect. A.,* 2008, **A64**, 112.

3. CrysAlis PRO version 1.171.37.35 (Rigaku Oxford Diffraction Ltd, Yarnton, Oxfordshire, UK, 2018.

4. G.M. Sheldrick, *Acta Cryst. Sect. C.,* 2015, **C71**, 3.

5. O.V. Dolomanov, L.J. Bourhis, R.J. Gildea, J.A.K. Howard, H. Puschmann, *J. Appl. Cryst.*, 2009, **42**, 339.

6. L.J. Farugia, *J. Appl. Cryst.* 2012, **45**, 849.

7. Persistence of Vision (TM) Raytracer v 3.6, Persistence of Vision Pty. Ltd. , Williamstown, Victoria, Australia.

8. D. M. King, F. Tuna, E. J. L. McInnes, J. McMaster, W. Lewis, A. J. Blake, S. T. Liddle, *Nat. Chem.,* 2013, **5**, 482.

9. D. M. King, P. A. Cleaves, A. J. Wooles, B. M. Gardner, N. F. Chilton, F. Tuna, W. Lewis, E. J. L. McInnes, S. T. Liddle, *Nat. Commun.,* 2016, **7**, 13773.

10. Gaussian 16, Revision A.03, M. J. Frisch, G. W. Trucks, H. B. Schlegel, G. E. Scuseria, M. A. Robb, J. R. Cheeseman, G. Scalmani, V. Barone, G. A. Petersson, H. Nakatsuji, X. Li, M. Caricato, A. V. Marenich, J. Bloino, B. G. Janesko, R. Gomperts, B. Mennucci, H. P. Hratchian, J. V. Ortiz, A. F. Izmaylov, J. L. Sonnenberg, D. Williams-Young, F. Ding, F. Lipparini, F. Egidi, J. Goings, B. Peng, A. Petrone, T. Henderson, D. Ranasinghe, V. G. Zakrzewski, J. Gao, N. Rega, G. Zheng, W. Liang, M. Hada, M. Ehara, K. Toyota, R. Fukuda, J. Hasegawa, M. Ishida, T. Nakajima, Y. Honda, O. Kitao, H. Nakai, T. Vreven, K. Throssell, J. A. Montgomery, Jr., J. E. Peralta, F. Ogliaro, M. J. Bearpark, J. J. Heyd, E. N. Brothers, K. N. Kudin, V. N. Staroverov, T. A. Keith, R. Kobayashi, J. Normand, K. Raghavachari, A. P. Rendell, J. C. Burant, S. S. Iyengar, J. Tomasi, M. Cossi, J. M. Millam, M. Klene, C. Adamo, R. Cammi, J. W. Ochterski, R. L. Martin, K. Morokuma, O. Farkas, J. B. Foresman, and D. J. Fox, Gaussian,

Inc., Wallingford CT, 2016.

11. TURBOMOLE V7.3 2015, a development of University of Karlsruhe and Forschungszentrum Karlsruhe GmbH, 2019, TURBOMOLE GmbH; available from turbomole.com..
12. J. P. Perdew, K. Burke, M. Ernzerhof, *Phys. Rev. Lett.,* 1996, **77**, 3865.
13. J. P. Perdew, M. Ernzerhof, K. Burke, *J. Chem. Phys.,* 1996, **105**, 9982.
14. X. Cao, M. Dolg, H. Stoll, *J. Chem. Phys.,* 2003, **118**, 487.
15. X. Cao, M. Dolg, *J. Mol. Struct. THEOCHEM,* 2004, **673**, 203.
16. W. Küchle, M. Dolg, H. Stoll, H. Preuss, *J. Chem. Phys.,* 1994, **100**, 7535.
17. T. H. Dunning, *J. Chem. Phys.,* 1989, **90**, 1007.
18. R. A. Kendall, T. H. Dunning, R. J. Harrison, *J. Chem. Phys.,* 1992, **96**, 6796.
19. D. E. Woon, T. H. Dunning, *J. Chem. Phys.,* 1993, **98**, 1358.
20. K. K. Pandey, P. Patidar, S. K. Patidar, R. Vishwakarma, *Spectrochim Acta A Mol Biomol Spectrosc,* 2014, **133**, 846.
21. I. Fdez. Galván, M. Vacher, A. Alavi, C. Angeli, F. Aquilante, J. Autschbach, J. J. Bao, S. I. Bokarev, N. A. Bogdanov, R. K. Carlson, et al., *J. Chem. Theory Comput.,* 2019, **15**, 5925.
22. Molpy, S. Vancoillie, 2017, available from github.com/steabert/molpy
23. R. F. Bader, *Atoms in Molecules: A Quantum Theory*, Clarendon Pr., 1990.
24. AIMAll (Version 19.10.12), Todd A. Keith, TK Gristmill Software, Overland Park KS, USA, 2019, available from aim.tkgristmill.com.
25. E. D. Glendening, J. K. Badenhoop, A. E. Reed, J. A. Carpenter, J. E. Bohmann, C. M. Morales, C. R. Landis, F. Weinhold, 2013.
